# Clinical Management of Poly‐D,L‐Lactic Acid Nodules: A Guideline With Diagnostic and Treatment Flowchart

**DOI:** 10.1111/jocd.70158

**Published:** 2025-03-31

**Authors:** Fabiano Nadson Magacho‐Vieira, Eliza Porciuncula Justo Ducati

**Affiliations:** ^1^ Magacho Institute for Health Education Fortaleza Ceará Brazil; ^2^ Department of Clinical, Aesthetic and Surgical Dermatology Batista Memorial Hospital Fortaleza Ceará Brazil; ^3^ ED Ultrassonografia Dermatológica São Paulo São Paulo Brazil

**Keywords:** adverse effects, collagen biostimulators, fillers, granuloma, nodule, poly‐D,L‐lactic acid

Safety is of critical importance in aesthetic treatments. There are currently two distinct varieties of poly‐lactic acid (PLA)‐based fillers available on the market: poly‐L‐lactic acid (PLLA) and poly‐D,L‐lactic acid (PDLLA). Both materials have been widely utilized in the field of aesthetic dermatology, primarily due to their capacity to induce a sub‐clinical inflammatory tissue response that causes an increase in the content of type I collagen at the injection site.

PDLLA has demonstrated a favorable safety profile [1]. However, although side effects are typically mild and transient, there are rare reports of more significant issues, such as nodules or granulomas. Unlike hyaluronic acid nodules, which can be readily dissolved with hyaluronidase injections that result in filler degradation, PDLLA nodules do not respond to straightforward enzymatic treatment, requiring a more intensive approach. Moreover, the biostimulatory nature of PDLLA (which promotes collagen production) may result in a more prolonged tissue response, potentially complicating the resolution of nodules and making it necessary to extend the treatment period. This can be a challenging scenario from a clinical perspective. Consequently, the formulation of a structured treatment‐focused guideline can assist dermatologists in optimizing treatment protocols for each individual case by following a series of diagnostic steps that ultimately lead to the formulation of corresponding therapeutic recommendations. Figure 1 illustrates a step‐by‐step guide for diagnosis and treatment, developed by integrating the authors' extensive clinical experience with a comprehensive review of the medical literature to create an evidence‐based, yet practical framework for managing these cases.

**FIGURE 1:**
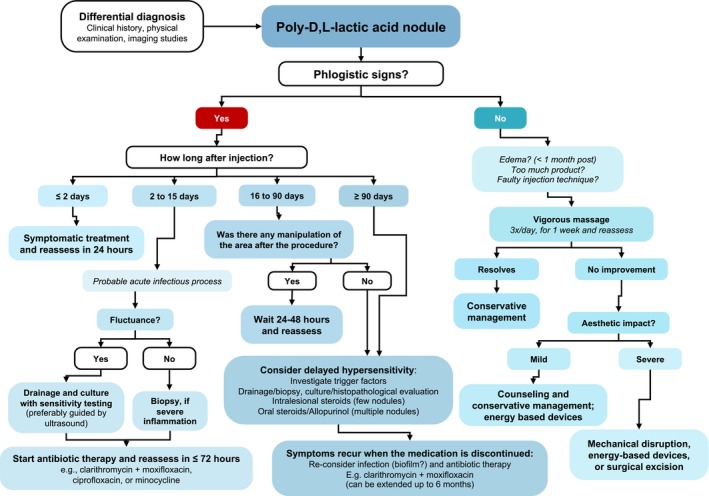
Flowchart illustrating a stepwise approach to managing poly‐D,L‐lactic acid (PDLLA) nodules, using questions and answers to navigate clinical decisions, with arrows guiding progression through clearly defined pathways. The authors note that some treatments in this approach rely on limited data and/or require further risk validation.

It is important to note that other pathologies that manifest as free, hardened nodules in the skin or subcutaneous tissue (such as cutaneous calcinosis, small epidermoid cysts, nodular basal cell carcinomas, or pseudolymphomas) may present in a similar manner to PDLLA nodules, thus underscoring the necessity for an initial differential diagnosis [2]. The multiplicity of potential etiologies highlights the importance of a comprehensive history, clinical examination, and diagnostic imaging for the appropriate management of these cases. Imaging is of significant value, particularly ultrasound (US), due to its superior accessibility and ability to characterize fillers and complications, while other techniques, such as magnetic resonance imaging (MRI) or computed tomography (CT), can provide valuable information in scenarios where sonographic findings are inconclusive or further anatomical detail is required [3]. In rare cases where history and imaging still render an inconclusive diagnosis, histopathological evaluation may be necessary.

As part of the consent process, patients must be informed about the potential risks and the importance of promptly notifying the clinician in the event of any changes in their symptoms. This is particularly critical for newer or experimental treatments, where uncertainties must be clearly communicated, and shared decision‐making emphasized. Ethical considerations also include ensuring that interventions are evidence‐based whenever possible and that documentation—such as photographic records and imaging findings—is maintained to support transparency and patient safety.

Filler‐associated nodules have a variety of etiologies, each presenting distinctive inflammatory signs, timelines, and diagnostic implications. Non‐inflammatory nodules result from localized accumulation or maldistribution of the product due to inadequate injection technique, inaccurate placement, or migration‐related factors such as muscle dynamics, gravitational pull, or post‐procedural massage [2, 4]. In contrast, inflammatory nodules (such as granulomas or abscesses due to infections) exhibit pronounced “phlogistic signs” including warmth, redness, tenderness, and swelling [5, 6]. In the latter case, the time of onset of symptoms can serve as a valuable indicator for diagnosis, subsequently influencing the course of treatment.

Early‐onset mild “phlogistic signs” like warmth, pain, and edema are common adverse effects following the injection of filler and often resolve within a few days. However, the persistence of these signs or symptoms, especially if they worsen over time, may indicate an infectious process. Infectious nodules frequently manifest within days to a few weeks following injection as tender, erythematous swellings. In some cases, these may progress into subcutaneous abscesses requiring drainage. Smaller abscesses can be more accurately diagnosed and drained with the help of high‐frequency ultrasound [7]. Antibiotic treatment should be initiated, ideally following the collection of material for a bacterial culture, especially in moderate to severe cases [8]. If no improvement is observed within 72 h, escalating the antibiotic should be considered.

Delayed hypersensitivity reactions to fillers are typically described as “angry red bumps,” [9] and usually appear 3 months to several years post‐injection. It is hypothesized that this is a T‐cell mediated hypersensitivity type IV late onset reaction that can progress to a specific granulomatous reaction [10]. These lesions typically occur in all areas treated with the offending filler. Treatment usually involves fluorinated corticosteroids (oral or intralesional) and broad‐spectrum antibiotics (especially if biofilm‐forming bacteria are suspected) in recalcitrant cases [6, 10, 11, 12]. Once again, high‐frequency ultrasound may prove advantageous in guiding the precise injection of the treatment [13].

Granulomatous nodules usually appear between six and 24 months post‐injection [6]. These nodules are typically firm and non‐fluctuant, gradually enlarging over time, and generally respond well to intralesional corticosteroid therapy [5]. Histopathological evaluation is required to confirm etiology and differentiate between infectious and granulomatous late onset reactions, as each type necessitates a distinct therapeutic approach based on its underlying pathology [4, 6].

For non‐inflammatory nodules, the timing of symptoms remains relevant for causal diagnosis, though it has a considerably diminished impact on treatment direction. These often present as small, firm nodules resulting from localized accumulation or maldistribution of the product due to inadequate injection technique, inaccurate placement, or migration‐related factors such as muscle dynamics, gravitational pull, or post‐procedural massage [2, 4, 5] within several months after injection, due to collagen formation. While firm and defined, these nodules lack overt inflammatory signs and respond poorly to corticosteroids.

The authors propose vigorous massage as a non‐invasive and often efficacious initial approach. Mechanical or pharmacochemical disruption of collagen stimulator nodules may also be considered. This can be carried out by performing a mechanical subcision with a needle while injecting sterile water, saline solution, 5‐fluorouracil, collagenase, hyaluronidase, and a range of other substances [14]. The use of ultrasound guidance may prove beneficial in ensuring the accuracy and precision of the approach [15]. However, based on the authors' experience, this approach is not particularly effective and can lead to additional setbacks. In cases where the nodule persists, surgical excision may be required [2, 4, 5].

It has recently been proposed that energy‐based devices may offer a potential mechanism for resolving PDLLA‐induced nodules. This proposal takes advantage of the lower glass transition temperature (at which the material assumes a soft and flexible, malleable state) of this material in comparison to other collagen stimulators. One case report [16] demonstrated that the application of a mono‐polar radio frequency directly to the nodules resulted in complete resolution within 24 h. This mechanism relies on heating PDLLA to temperatures between 38°C and 39°C, combined with manual manipulation, such as massage, to facilitate non‐invasive reshaping of the nodule. Although promising, it is important to note that this approach is based on limited data and still requires further validation, as well as an assessment of potential risks.

An additional consideration in the management of patients with adverse outcomes is the choice between interventional and conservative approaches. There can be a temptation to respond immediately when an adverse reaction occurs. However, the financial, emotional, and physical toll of overtreatment is significant. It is essential to carefully evaluate whether intervention is truly necessary for minor issues, especially those with minimal aesthetic impact. Avoiding unnecessary screening, diagnostics, or non‐beneficial procedures (especially surgical ones) not only reduces costs and patient burden but also helps prevent the risk of obfuscating, prolonging, or even exacerbating the original complaint. These situations provide the opportunity for shared decision‐making, allowing physicians and patients to discuss tailored benefits and risks to ensure that the patient's choices align with their values [17].

In conclusion, while PDLLA‐based fillers are highly effective in promoting collagen stimulation, the potential for complications such as nodule formation underscores the importance of precise diagnosis and individualized management strategies. Differentiating between inflammatory and non‐inflammatory nodules (as well as other potential etiologies) requires a thorough clinical evaluation complemented by imaging techniques when appropriate. Although long‐term studies are limited, anecdotal evidence and the authors' experience suggest that recurrence is more likely when the inflammatory response is not adequately controlled. While treatment options for PDLLA nodules are well documented, their long‐term outcomes and recurrence rates remain unclear.

The multitude of mechanisms and available treatment options can complicate clinical decision‐making, highlighting the need for a structured, problem‐based approach. The proposed diagnostic and therapeutic flow chart provides clinicians with a guided framework to navigate these complexities, ensuring that management decisions are both systematic and tailored to the patient's specific presentation. Further research, including multicenter studies and clinical trials, is essential to standardize management strategies and validate this guideline, ensuring safe and optimal outcomes for patients.

## Author Contributions

The authors confirm contribution to this article as follows. Conception, design, and draft preparation: F.N.M‐V. Critical review for important intellectual content: E.P.J.D. All authors reviewed, approved, and agreed to be accountable for all aspects of the final version of this article.

## Ethics Statement

The authors have nothing to report.

## Consent

There are no photos or patient personal information in this article.

## Conflicts of Interest

F.N.M.‐V. serves as medical director for Dermadream Corporation. E.P.J.D. is a regular speaker for Dermadream Corporation.

## Data Availability

Data sharing not applicable to this article as no datasets were generated or analysed during the current study.

## References

[1] Y. A. No , J. Seok , M. Y. Hyun , et al., “Long‐Term (24‐Month) Safety Evaluation of Poly‐DL‐Lactic Acid Filler Injection for the Nasolabial Fold: A Multicenter, Open, Randomized, Evaluator‐Blind, Active‐Controlled Design,” Plastic and Reconstructive Surgery 135, no. 6 (2015): 1074e–1075e, 10.1097/PRS.0000000000001247. 25724056

[2] F. N. Magacho‐Vieira and A. P. Santana , “Displacement of Hyaluronic Acid Dermal Filler Mimicking a Cutaneous Tumor: A Case Report,” Clinical, Cosmetic and Investigational Dermatology 16 (2023): 197–201, 10.2147/CCID.S398014. 36711075 PMC9882410

[3] X. Wortsman , “Identification and Complications of Cosmetic Fillers: Sonography First,” Journal of Ultrasound in Medicine 34, no. 7 (2015): 1163–1172, 10.7863/ultra.34.7.1163. 26112618

[4] T. R. Kwon , S. W. Han , I. K. Yeo , et al., “Biostimulatory Effects of Polydioxanone, Poly‐ d, l Lactic Acid, and Polycaprolactone Fillers in Mouse Model,” Journal of Cosmetic Dermatology 18, no. 4 (2019): 1002–1008, 10.1111/jocd.12950. 30985064

[5] K. M. Perez Willis and G. R. Ramirez , “Granuloma After the Injection of Poly‐D,L‐Lactic Acid (PDLLA) Treated With Triamcinolone,” Case Reports in Dermatological Medicine 2024 (2024): 6544506, 10.1155/2024/6544506. 38698953 PMC11065485

[6] G. W. Hong , H. Hu , K. Chang , et al., “Review of the Adverse Effects Associated With Dermal Filler Treatments: Part I Nodules, Granuloma, and Migration,” Diagnostics 14, no. 15 (2024): 1640, 10.3390/diagnostics14151640. 39125515 PMC11311355

[7] A. G. Nicola , M. O. Pricop , and B. Ramos‐Medina , “Clinical Management With High‐Frequency Ultrasound of Recurrent Submental Abscess Formation After Filler Placement: Bacterial Contamination or Immune‐Mediated Adverse Event?,” Cureus 16, no. 4 (2024): e58878, 10.7759/cureus.58878. 38659708 PMC11040211

[8] E. Davies , D. Vaghela , C. Convery , L. Walker , and G. Murray , “Guideline for the Prevention, Diagnosis, and Management of Acute Bacterial Soft Tissue Infections Following Nonsurgical Cosmetic Procedures,” Journal of Clinical and Aesthetic Dermatology 14, no. Suppl 1 (2021): S29–S35. PMC8562943 34980965

[9] R. S. Narins , M. Jewell , M. Rubin , J. Cohen , and J. Strobos , “Clinical Conference: Management of Rare Events Following Dermal Fillers—Focal Necrosis and Angry Red Bumps,” Dermatologic Surgery 32, no. 3 (2006): 426–434, 10.1111/j.1524–4725.2006.32086.x. 16640693

[10] W. Baranska‐Rybak , J. V. Lajo‐Plaza , L. Walker , and N. Alizadeh , “Late‐Onset Reactions After Hyaluronic Acid Dermal Fillers: A Consensus Recommendation on Etiology, Prevention, and Management,” Dermatologic Therapy 14, no. 7 (2024): 1767–1785, 10.1007/s13555-024-01202-3. PMC11265052 38907876

[11] Y. L. Zhang , Z. S. Sun , W. J. Hong , Y. Chen , Y. F. Zhou , and S. K. Luo , “Biofilm Formation Is a Risk Factor for Late and Delayed Complications of Filler Injection,” Frontiers in Microbiology 14 (2024): 1297948, 10.3389/fmicb.2023.1297948. 38260874 PMC10800873

[12] C. Convery , E. Davies , G. Murray , and L. Walker , “Delayed‐Onset Nodules (DONs) and Considering Their Treatment Following Use of Hyaluronic Acid (HA) Fillers,” Journal of Clinical and Aesthetic Dermatology 14, no. 7 (2021): E59–E67. 34840652 PMC8570356

[13] G. Munhoz , F. A. Cavallieri , L. K. de Almeida Balassiano , et al., “Sterile Abscess due to Hyaluronic Acid: A New Diagnosis and a Proposal for Treatment‐A Series of Eight Cases,” Journal of Cosmetic Dermatology 21, no. 11 (2022): 5562–5568, 10.1111/jocd.15135. 35638403

[14] A. D. McCarthy , J. van Loghem , K. A. Martinez , S. B. Aguilera , and D. Funt , “A Structured Approach for Treating Calcium Hydroxylapatite Focal Accumulations,” Aesthetic Surgery Journal 44, no. 8 (2024): 869–879, 10.1093/asj/sjae031. 38366791 PMC11333958

[15] F. A. Cavallieri , L. K. A. Balassiano , G. Munhoz , M. F. Tembra , and X. Wortsman , “Ultrasound in Aesthetics: Filler and Non‐Filler Applications,” Seminars in Ultrasound, CT, and MR 45, no. 3 (2024): 251–263, 10.1053/j.sult.2023.11.005. 38072289

[16] S. B. Seo , J. Wan , and K. H. Yi , “Energy‐Based Device Management of Nodular Reaction Following Poly‐D,L‐Lactic Acid Injection for Tear Trough Rejuvenation,” Journal of Cosmetic Dermatology 24 (2025): e16575, 10.1111/jocd.16575. 39283001 PMC11743236

[17] J. Herzstein and M. Ebell , “Improving Quality by Doing Less: Overtreatment,” American Family Physician 91, no. 5 (2015): 289–291. 25822384

